# Integrative Analysis of Cytotoxic Lymphocytes‐Mediated Tumour Killing Related Genes Reveals LDHA as a Novel Glioblastoma Therapeutic Target

**DOI:** 10.1111/jcmm.71054

**Published:** 2026-02-13

**Authors:** Shaojie Yu, Xudong Li, Ji Wu, Minjie Wang, Zihan Gong, Baowen Zhang, Haojun Shi, Xuan Wang, Wei Xiang

**Affiliations:** ^1^ Department of Neurosurgery Union Hospital, Tongji Medical College, Huazhong University of Science and Technology Wuhan People's Republic of China; ^2^ Hubei Key Laboratory of Biological Targeted Therapy Union Hospital, Tongji Medical College, Huazhong University of Science and Technology Wuhan People's Republic of China; ^3^ Department of Neurosurgery Zhongnan Hospital of Wuhan University Wuhan People's Republic of China; ^4^ Department of Radiology Union Hospital, Tongji Medical College, Huazhong University of Science and Technology Wuhan People's Republic of China

**Keywords:** cytotoxic lymphocyte‐mediated tumour killing, glioblastoma, immunotherapy, LDHA, prognostic signature

## Abstract

Glioblastoma (GBM) features profound immunosuppression and metabolic reprogramming that undermine immunotherapy. We sought biomarkers that capture cytotoxic lymphocyte activity and nominate tractable targets. Using TCGA, we quantified NK/CD8+ T‐cell infiltration (CIBERSORTx), identified differentially expressed genes and derived a three‐gene prognostic index (CTLsTKPI: LDHA, TNIP1, PTPN2) via Cox and LASSO. Performance was assessed in REMBRANDT, CGGA325 and CGGA693, and CTLsTKPI was incorporated into a multivariable nomogram alongside clinical variables. We examined associations with immune checkpoints, tumour mutational burden and stemness, and performed functional studies of LDHA in GBM cells, CAR‐NK92 assays and an orthotopic mouse model. We detected 3195 DEGs versus normal brain; 119 tracked with cytotoxic infiltration. CTLsTKPI stratified survival in TCGA (AUCs 0.694/0.656/0.819 at 1/2/3 years) and validated across cohorts; the nomogram outperformed individual predictors (AUC 0.78). High CTLsTKPI aligned with checkpoint pathway activation, Treg/macrophage enrichment and reduced activated NK/CD8^+^ T cells. LDHA was the dominant adverse component, linked to glycolysis; its overexpression enhanced proliferation, migration and invasion, while lactate impaired CAR‐NK cytotoxicity. In vivo, LDHA inhibition (GNE‐140) synergised with PD‐1 blockade to reduce tumour burden and prolong survival. CTLsTKPI provides a clinically actionable biomarker, and LDHA represents a rational therapeutic target.

**Abbreviations**
AUC: area under the curve
BLI: bioluminescent imaging
CTLsTK: cytotoxic lymphocyte–mediated tumour‐killing
DEGs: differentially expressed genes
FDR: false discovery rate
GBM: glioblastoma
GO: gene ontology
ICIs: immune checkpoint inhibitors
LDHA: lactate dehydrogenase A
MCT1: monocarboxylate transporter 1
OS: overall survival
ROC: receiver operating characteristic
TMB: tumour mutational burden
TME: tumour microenvironment

## 1 Introduction

Glioblastoma (GBM) is among the most aggressive and lethal primary malignancies of the central nervous system, characterised by marked molecular and cellular heterogeneity, rapid progression and resistance to radiotherapy and chemotherapy [1, 2]. Despite advances in surgery, radiotherapy and chemotherapy, the median overall survival (OS) remains under 15 months, with early postoperative recurrence and progression common [3]. At the systems‐biology level, core cancer hallmarks—especially metabolic reprogramming and immune evasion—drive tumour evolution and therapeutic resistance. Aerobic glycolysis (the Warburg effect) sustains high glycolytic flux and excessive lactate production, acidifying the tumour microenvironment (TME) and fostering malignant behaviour [4]. In GBM, a profoundly immunosuppressive TME poses a central barrier to effective immunotherapy; impaired recruitment and diminished effector function of cytotoxic lymphocytes (NK and CD8^+^ T cells) facilitate immune escape and disease progression [5, 6].

Immune checkpoint inhibitors (ICIs) targeting PD‐1/PD‐L1 and CTLA‐4 have produced clinical benefit in several cancers, yet their efficacy in GBM remains limited [7, 8]. Contributing barriers include a profoundly immunosuppressive TME, restricted drug delivery across the blood–brain barrier and impaired effector lymphocyte function. Accordingly, single‐agent ICIs are unlikely to substantially improve outcomes in GBM, underscoring the need for predictive biomarkers and rational combination strategies [2, 9]. In this setting, conventional indicators such as PD‐L1 expression and tumour mutational burden have shown limited predictive value, whereas transcriptomic biomarkers that more directly capture cytotoxic lymphocyte activity and the broader immune contexture may better guide prognosis and immunotherapy selection [10, 11, 12, 13].

To address these limitations, we systematically profiled transcriptional correlates of cytotoxic lymphocytes in GBM and across independent cohorts, identifying differentially expressed genes (DEGs) tightly associated with NK/CD8^+^ T cells infiltration. From these, we curated a cytotoxic lymphocyte–mediated tumour‐killing (CTLsTK) gene set that stratifies tumours by immune‐infiltration patterns and nominates candidates with adverse prognostic associations, providing a basis for robust prognostic modelling and mechanistic dissection of immune escape [14, 15]. We then derived a three‐gene signature (LDHA, TNIP1, PTPN2) and computed a composite risk score (CTLsTKPI) that reproducibly separates patients into high‐ and low‐risk groups with significant survival differences across validation cohorts; integrating CTLsTKPI with clinicopathologic variables in a nomogram further improved individualised survival estimation. Finally, CTLsTKPI correlated with immune‐checkpoint activity and tumour mutational burden (TMB), supporting its potential to inform immunotherapy selection and underscoring its translational relevance in GBM.

Within this three‐gene signature, LDHA (lactate dehydrogenase A) emerges as a nexus linking metabolism and immunity. LDHA expression inversely correlates with NK/CD8^+^ T cells infiltration and positively with stromal/immune scores in GBM; pan‐cancer analyses likewise show widespread LDHA upregulation across malignancies [16]. Pathway enrichment implicates JAK–STAT and Toll‐like receptor signalling—features consistent with an immunosuppressive state—supporting LDHA as a putative driver of immune suppression and an actionable node on the immunometabolic axis [17, 18]. Lactate accumulation driven by LDHA impairs antitumour immunity and parallels canonical checkpoints (PD‐1, CTLA‐4), functioning as a ‘metabolic checkpoint’ [19, 20]. In GBM, particularly in IDH‐wild‐type disease, LDHA‐mediated lactate production is increased and monocarboxylate transporter 1 (MCT1) mediates transmembrane lactate transport [21, 22, 23]. Clinically, higher serum lactate associates with higher grade, elevated Ki‐67 index and larger tumour volume, indicating poorer prognosis [24, 25, 26]. These observations reinforce the rationale for therapeutically targeting lactate‐driven adaptive metabolic programmes in GBM.

Functionally and mechanistically, our findings substantiate this model. In vitro, LDHA overexpression markedly enhanced GBM cell proliferation, colony‐formation, migration and invasion, whereas gene silencing produced the opposite effects [27]. Mechanistically, LDHA‐driven lactate accumulation profoundly attenuated CAR‐NK‐mediated cytotoxicity. In an orthotopic GBM model, combined administration of the LDHA inhibitor GNE‐140 and an anti‐PD‐1 antibody reduced tumour burden and prolonged survival, providing proof of concept that targeting LDHA can potentiate checkpoint blockade in GBM [28, 29].

Collectively, by integrating multi‐cohort transcriptomics with immune context, we derived a prognostic index that robustly predicts survival and immunotherapy response in GBM. Within this model, LDHA is the top‐weighted risk gene, mechanistically linking lactate metabolism to immune suppression along the immunometabolic axis. These findings establish CTLsTKPI as a clinically actionable tool for risk stratification and treatment planning and nominate LDHA as a tractable target for metabolism–immunity combination therapy in GBM.

## 2 Materials and Methods

### 2.1 Data Acquisition and Preprocessing

GBM transcriptomic and clinical data were obtained from The Cancer Genome Atlas (TCGA, https://www.cancer.gov/tcga/), the Chinese Glioma Genome Atlas (CGGA, https://www.cgga.org.cn/) and the REMBRANDT database (https://wiki.cancerimagingarchive.net/display/Public/REMBRANDT). The study included the CGGA325‐GBM (*n* = 135), CGGA693‐GBM (*n* = 146), REMBRANDT (*n* = 237) and TCGA‐GBM (*n* = 211) cohorts. Samples with missing or ambiguous clinical annotations were excluded. TCGA served as the training set, and CGGA325, CGGA693 and REMBRANDT were used as external validation cohorts.

### 2.2 Software Versions and Parameter Settings

The glmnet (v4.1–10) package was applied for the LASSO‐Cox regression model, with α = 1 and λ selected at lambda.min via 10‐fold cross‐validation. Gene set enrichment analysis was performed using fgsea (v1.26.0), with minSize = 15, maxSize = 500 and 1000 permutations. Immune cell deconvolution was conducted using CIBERSORTx (Docker 1.7), with 1000 permutations and the LM22 signature matrix. For batch effect removal, we employed sva/ComBat (v3.46.0) using parametric empirical Bayes with default settings. Additionally, false discovery rate (FDR) corrections (Benjamini–Hochberg) were uniformly applied across all analyses to control for multiple testing.

### 2.3 Differentially Expressed CTLsTK‐Related Genes

Differential expression between 179 GTEx normal brain samples and TCGA GBM tumours was analysed with limma, using Benjamini–Hochberg correction (|log2FC| > 1; FDR < 0.05) [30]. Before analysis, low‐abundance genes were filtered and GTEx/TCGA batch effects were corrected with ComBat (sva). Immune infiltration was estimated with CIBERSORTx, and Spearman correlations between gene expression and NK/CD8^+^ T‐cell infiltration identified 119 CTLsTK‐related genes (|r| > 0.3; FDR < 0.05) [31]. Details are provided in Table S1. Samples were dichotomised at median infiltration and these genes were visualised using pheatmap (row z‐scores; hierarchical clustering with group annotations). The overlap of DEGs with CTLsTK‐related genes defined the differentially expressed CTLsTK set.

### 2.4 Pathway and Function Enrichment Analysis

To explore the potential biological roles of CTLsTK‐associated DEGs, functional enrichment analyses were performed using the R package ‘clusterProfiler’. Gene Ontology (GO) terms and KEGG signalling pathways were analysed, applying a FDR threshold of 5%. Enrichments were considered statistically significant when *p* < 0.05.

### 2.5 CTLsTK‐Related Gene Prognostic Signature Construction and Validation

The univariate Cox proportional hazards regression (*p* < 0.05) was first used to evaluate associations between CTLsTK‐related DEGs and OS in GBM, yielding four prognostic DEGs. The candidate features were then reduced and an optimal signature was derived via LASSO–Cox regression, with the penalty parameter selected at lambda.min using the R package glmnet [32]. For each patient, a CTLsTK score was computed as the linear predictor:

$$\text{CTLsTK}=\sum_{i=1}^{3}\left(\mathrm{\beta i}\times \mathrm{Ei}\right)$$

where βi represents the risk coefficient and Ei represents the expression level of each gene. The Kaplan–Meier curves and log‐rank tests were generated using survival and survminer to assess the relationship between the CTLsTK score and OS.

### 2.6 Establishment of the Prognostic Nomogram

To determine the optimal cutoff values for stratifying patients into high‐ and low‐expression groups in survival analyses, we used the surv_cutpoint function from the survminer R package, which identifies the threshold that maximises the separation between survival curves based on maximally selected rank statistics. Survival differences were subsequently evaluated using Kaplan–Meier and Cox proportional hazards models. The CTLsTKPI risk‐score formula (model coefficients) was fixed based on the TCGA training cohort; for each cohort, patients were dichotomised into high‐ and low‐risk groups using a cohort‐specific optimal cutpoint identified by surv_cutpoint.

To develop a prognostic nomogram, clinical variables including sex and age were integrated with CTLsTKPI based on the results of multivariable stepwise Cox regression analysis. The nomogram was constructed using the ‘rms’ and ‘regplot’ packages in R. The predictive accuracy of the model was evaluated by generating time‐dependent ROC curves using the ‘timeROC’ package, with the AUC calculated to quantify model performance. Additionally, calibration curves were plotted using the ‘rms’ package to assess the agreement between predicted survival probabilities and observed outcomes, providing a measure of the nomogram's reliability in survival prediction.

### 2.7 Cell Lines and Cell Culture

Human glioblastoma cell lines U251 and A172 were obtained from the American Type Culture Collection (ATCC, USA). Cells were cultured in DMEM supplemented with 10% fetal bovine serum (FBS; Gibco, USA) and penicillin–streptomycin (10 mg/mL; Beyotime, China) at 37°C in a humidified incubator with 5% CO_2_. Cells were used within 6 months of thawing, and routine mycoplasma testing confirmed the absence of contamination.

The NK92‐GFP‐EGFRvIII·CAR (CD28·CD3ζ) cells were purchased from Vigen Biotechnology (Zhenjiang, China) (Catalogue: VGC‐0011‐017S). These CAR‐NK cells were cultured in MEMα complete medium containing 0.2 mM inositol, 0.1 mM β‐mercaptoethanol, 0.02 mM folic acid, 200 U/mL recombinant IL‐2 (Peprotech), 12.5% horse serum (Sigma), 12.5% FBS (Gibco), 100 U/mL penicillin and 0.1 mg/mL streptomycin (Gibco). For preactivation and expansion, recombinant human IL‐2 was supplemented in the medium, and half of the IL‐2‐containing medium was replaced with fresh medium three times a week. The cells were maintained at a density of 1–3 × 10^7^ cells/mL, with fresh complete medium added as needed to maintain cell growth. Preactivated NK cells were used for cytotoxicity assays after 3 days of activation and within 2 weeks of activation (from Day 3 to Day 14).

### 2.8 Lentivirus Infections

LDHA overexpression plasmid (pLVX‐AcGFP‐N1) and LDHA‐specific shRNAs were obtained from Shanghai Jikai Gene Chemical Technology (Shanghai, China). The shRNA sequences (5′ → 3′) were: NC, TTCTCCGAACGTGTCACGT; sh1, GGGACTGATAAAGATAAGGAACA; sh2, TTGAAGAAGAGTGCAGATACACT. U251 and A172 cells were transduced with the corresponding lentiviruses and, 48 h later, selected with puromycin (2 μg/mL; Sigma‐Aldrich, USA) for 1–2 weeks to establish stable lines. LDHA overexpression or knockdown efficiency was verified by RT‐qPCR and Western blotting.

### 2.9 Western Blotting

Cells were lysed in ice‐cold RIPA buffer supplemented with a protease inhibitor cocktail (Roche, Mannheim, Germany) for 30 min on ice. Lysates were clarified by centrifugation at 12,000 rpm for 15 min at 4°C and protein concentrations were determined by BCA assay (Thermo Fisher Scientific). Equal protein (25 μg per lane) was resolved by SDS‐PAGE and transferred to PVDF membranes (Millipore). Membranes were blocked in 5% skim milk in TBST (TBS with 0.1% Tween‐20) for 1.5 h at room temperature, incubated overnight at 4°C with primary antibodies, washed in TBST and then incubated with HRP‐conjugated secondary antibodies for 1.5 h at room temperature. Signals were developed using enhanced chemiluminescence (ECL; Thermo Fisher Scientific) and captured on an Amersham Imager 600 (GE Healthcare). Primary antibodies: LDHA (19987–1‐AP, Proteintech, China) and GAPDH (Ab8226, Abcam, USA).

### 2.10 CCK‐8 Assay

Cell viability was assessed using the CCK‐8 assay kit (K1018, Apexbio, USA). Cells were seeded in 96‐well plates at 1 × 10^4^ cells/well. At designated time points (0, 24, 48 and 72 h), 10 μL of CCK‐8 reagent was added to each well and incubated at 37°C for 2 h. Absorbance at 450 nm was recorded with a microplate reader (Bio‐Rad, Hercules, CA, USA).

### 2.11 EdU Assays

GBM cells were seeded into 48‐well plates and cultured until they reached approximately 50%–80% confluence. Cell proliferation was then assessed using the BeyoClick EdU Cell Proliferation Kit with Alexa Fluor 594 (C0078L, Beyotime) following the manufacturer's protocol. EdU‐positive cells were visualised and quantified using a fluorescence microscope (Nexcope NE930, Ningbo, China).

### 2.12 Colony‐Formation Assay

For the colony formation assay, 400 cells were plated into 6‐well plates and cultured with medium refreshed every 4 days. Visible colonies typically appeared after 2–3 weeks of incubation, at which point they were fixed in 4% paraformaldehyde for 15 min. The colonies were subsequently stained with 0.1% crystal violet for 30 min, imaged and quantified based on both their size and number.

### 2.13 Cell Migration and Invasion Assays

Transwell migration and invasion assays were performed using inserts that were either uncoated (migration) or Matrigel‐coated (invasion). Serum‐starved GBM cells (1 × 10^4^) suspended in 200 μL serum‐free medium were seeded into the upper chamber; 600 μL complete medium containing 20% FBS was added to the lower chamber as a chemoattractant. After 36 h, nonmigrated cells on the upper membrane surface were removed with a cotton swab. Cells on the underside were fixed in 4% paraformaldehyde for 15 min, stained with 0.1% crystal violet for 30 min and quantified in five randomly selected fields per insert.

### 2.14 Xenograft Tumour Model

All animal procedures were approved by the Institutional Animal Care and Use Committee of Tongji Medical College and conducted in accordance with the NIH Guide for the Care and Use of Laboratory Animals (approval no. [2023] IACUC 3807). C57BL/6 mice were randomly assigned to four groups and anaesthetised. Using a stereotaxic frame, 5 μL of GBM cell suspension (5 × 10^5^ cells; with the indicated treatment) was injected intracranially 2 mm lateral and 2 mm anterior to the bregma at a depth of 3 mm. Tumour growth was monitored by bioluminescence imaging (Bruker, Billerica, MA, USA). Neurological signs, tumour burden and overall survival were recorded for each group.

### 2.15 Immunohistochemistry (IHC) Assay and Terminal Deoxynucleotidyl Transferase dUTP Nick End Labelling (TUNEL) Assay

Formalin‐fixed (4% paraformaldehyde), paraffin‐embedded specimens from human and mouse tissues were cut into serial 6‐μm sections. Sections were processed for IHC with primary antibodies against LDHA (Proteintech, 19,987–1‐AP; China), CD56 (Proteintech, 14,255–1‐AP; China) and CD8 (Abcam, ab237709; USA). Slides were examined by bright‐field microscopy (Leica, Germany), and immunoreactivity was quantified as the percentage of marker‐positive cells.

TUNEL staining was performed using the In Situ Cell Death Detection Kit, POD (Roche, Switzerland) in accordance with the manufacturer's instructions. Images were acquired on an Olympus FSX100 microscope (Olympus, Tokyo, Japan).

### 2.16 Statistical Analysis

Statistical analyses were performed in GraphPad Prism 8 (GraphPad Software). Data are presented as mean ± SD. Associations were evaluated by Spearman's rank correlation. Two‐group comparisons used two‐sided Student's *t*‐tests. Mouse survival was analysed by Kaplan–Meier with the log‐rank test. *p* < 0.05 was considered statistically significant. All experiments were independently repeated at least three times.

## 3 Results

### 3.1 Identification of CTLsTK‐Related DEGs

An overview of the workflow is shown in Figure 1A. In the TCGA GBM cohort, CIBERSORTx R package was used to quantify NK/CD8^+^ T cells infiltration. Differential analysis versus healthy brain identified 3195 DEGs (|log_2_FC| > 1; FDR < 0.05). Of these, 119 genes correlated with NK/CD8^+^ T infiltration (17 positively, 102 negatively), and hierarchical clustering of these genes clearly separated high‐ and low‐infiltration tumours (Figure 1B). Integrating survival data highlighted five negatively correlated, adverse prognostic genes—VEGFA, TNIP1, LDHA, BCKDK and PTPN2 (Figure 1C)—which exhibited strong co‐expression (Figure 1D) and were markedly upregulated in GBM relative to normal brain tissues (Figure 1E). Immune infiltration profiling showed positive associations with neutrophils and resting NK cells but inverse relationships with activated NK cells and CD8^+^ T cells (Figure 1F); and LDHA displayed the strongest negative correlation with cytotoxic lymphocyte infiltration (Figure 1G). Collectively, these 119 CTLsTK‐related DEGs delineate molecular differences between GBM with high versus low NK/CD8^+^ T cells infiltration, offering insight into mechanisms of an immunosuppressive TME.

**FIGURE 1:**
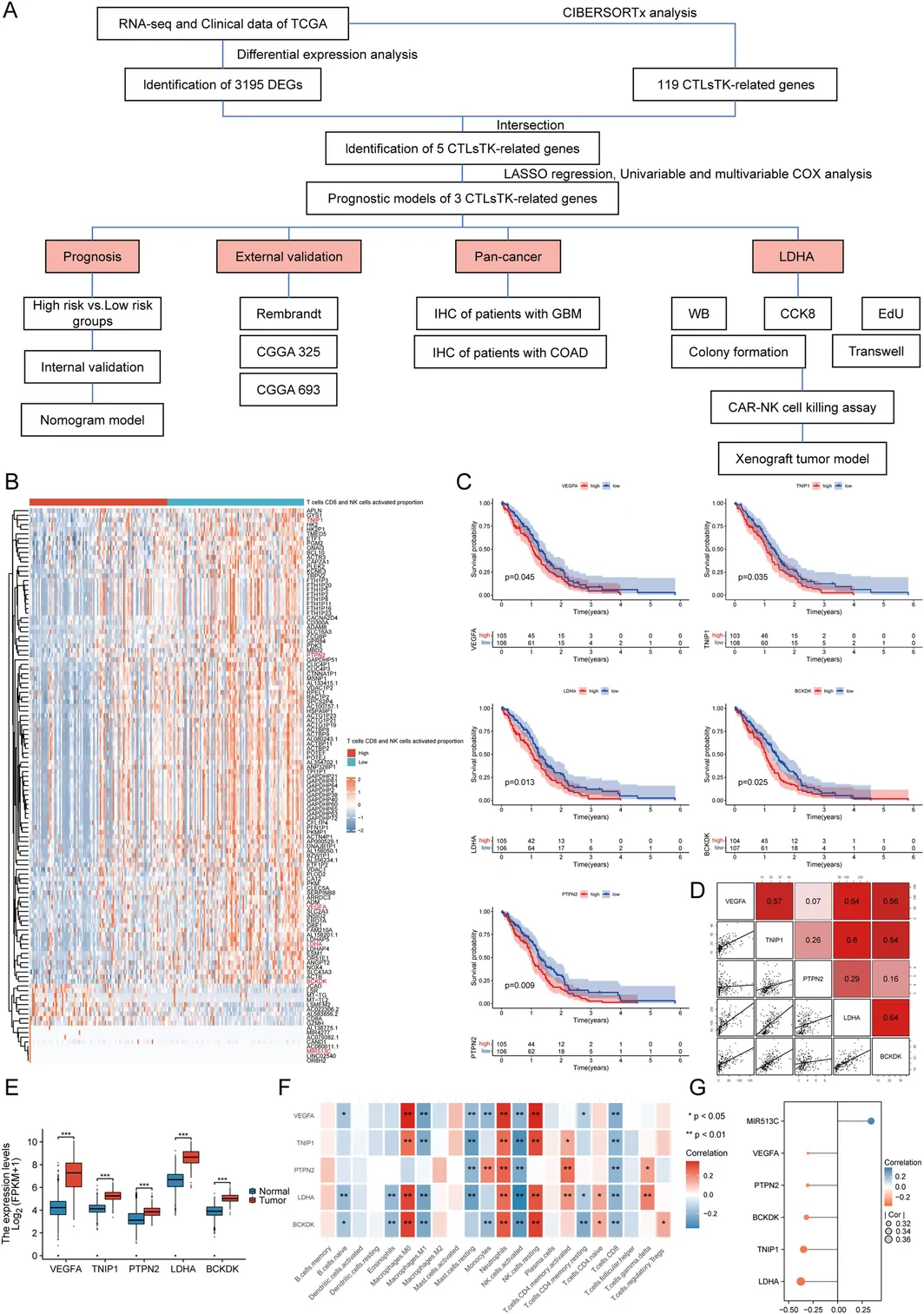
Identification of CTLsTK‐related DEGs. (A) The flowchart of this study. (B) Heatmap of gene expression stratified by high versus low NK/CD8^+^ T cells infiltration. (C) Kaplan–Meier survival curves of VEGFA, TNIP1, LDHA, BCKDK and PTPN2 according to expression levels. (D) Correlation analysis among the above five genes. (E) Differential expression of the five genes between GBM and normal tissues in TCGA. (F) Correlations between the five genes and immune cells infiltration. (G) Lollipop plot depicting associations of the five genes with NK/CD8^+^ T cells infiltration, with LDHA ranking as the most negative. *p < 0.05, **p < 0.01, ***p < 0.001, ****p < 0.0001.

### 3.2 CTLsTK‐Related Prognostic Signature Construction

To develop a prognostic biomarker for GBM based on CTLsTK‐associated differentially expressed genes, we established a risk score prediction model. In the TCGA training cohort, univariate Cox proportional hazards analysis (*p* < 0.05) identified four CTLsTK‐related DEGs associated with overall survival (OS). Subsequently, LASSO regression analysis with cross‐validation was applied to determine the optimal model parameters, resulting in a three‐gene signature comprising TNIP1, PTPN2 and LDHA (Figure 2A,B). The CTLsTK‐derived prognostic index (CTLsTKPI) was defined as: CTLsTKPI = (0.0552283605 × expression of LDHA) + (0.0194419232 × expression of TNIP1) + (0.0161917634 × expression of PTPN2). Further univariate Cox regression analysis revealed that LDHA showed the strongest association with poor OS. A forest plot summarises hazard ratios and 95% CIs for the three genes and the composite score (Figure 2C,D). Moreover, Pan‐cancer interrogation further revealed marked upregulation of these genes in DLBC, GBM, KIRC, PAAD and CHOL, suggesting their potential biological relevance and prognostic value in a broad spectrum of malignancies (Figure 2E). Overall, the three‐gene CTLsTK signature provides an effective risk score that stratifies GBM patients into low‐risk and high‐risk groups and demonstrates reliable prognostic utility.

**FIGURE 2:**
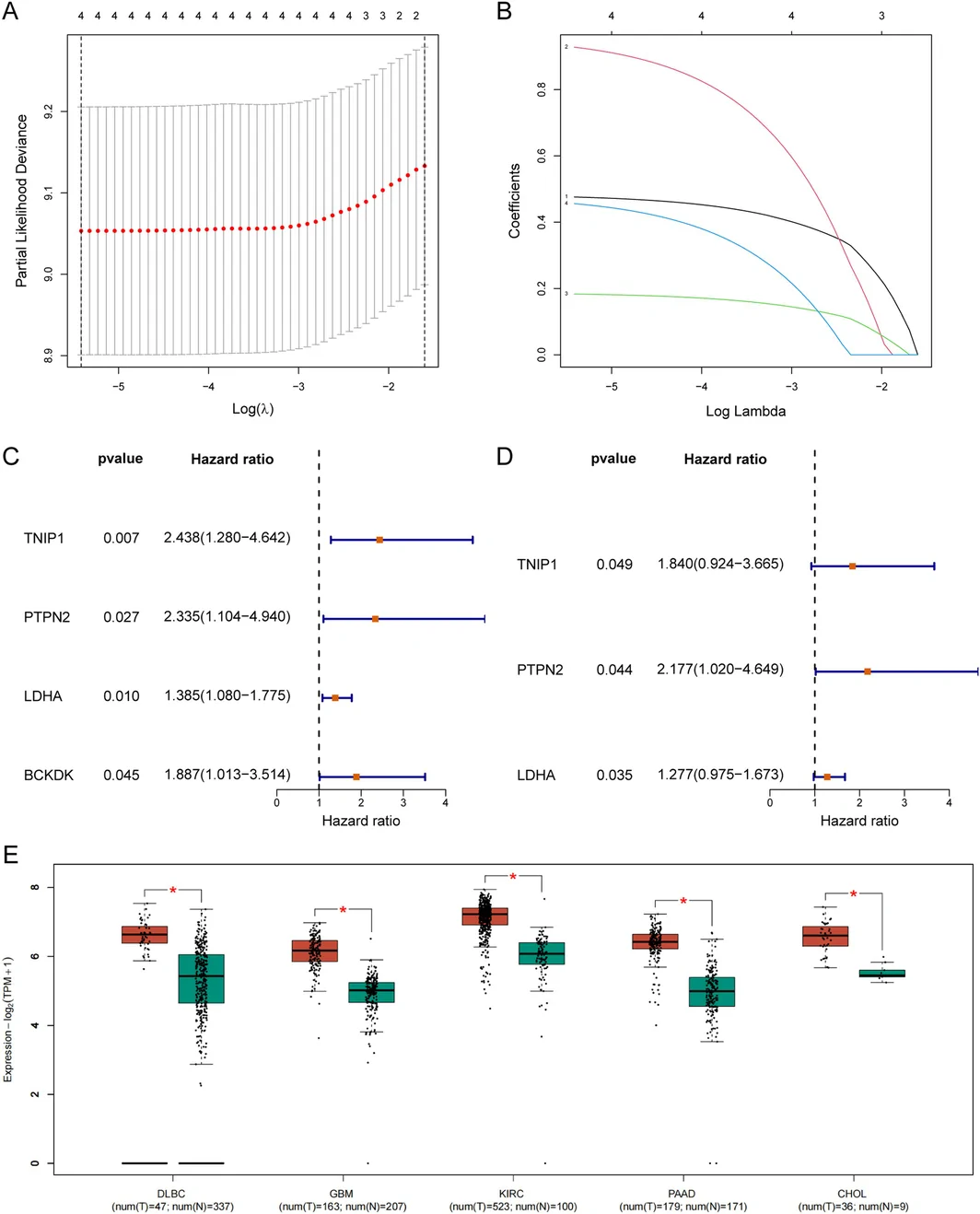
Construction and validation of an immune‐related gene signature. LASSO regression identifying signature genes. (B) Cross‐validation of the signature to select the optimal λ. (C‐D) Forest plots of univariate (C) and multivariate (D) Cox regression displaying hazard ratios and 95% confidence intervals for each TTKPI‐related gene. (E) Pan‐cancer analysis demonstrating elevation of the CTLsTK signature in DLBC, GBM, KIRC, PAAD and CHOL.

### 3.3 Train and Validate the Risk Score Model for GBM Patients

Building on the three‐gene CTLsTK signature, we derived a composite risk score that stratifies GBM patients into low‐risk and high‐risk categories and serves as a putative prognostic biomarker. Overall survival (OS) analyses showed that higher CTLsTKPI values were associated with shorter survival (Figure 3A,B). In the TCGA cohort, receiver operating characteristic (ROC) curves demonstrated good discrimination for OS with area under the curve (AUC) values of 0.694, 0.656 and 0.819 at 1, 2 and 3 years, respectively (Figure 3C). Model robustness was then examined in three independent cohorts—REMBRANDT (*n* = 237), CGGA‐325 (*n* = 135) and CGGA‐693 (*n* = 146). Using the same CTLsTKPI risk‐score formula, patients in each dataset were separated into high‐ and low‐risk groups using cohort‐specific optimal cutpoints (Figure S1A–C). Kaplan–Meier analyses confirmed significantly poorer OS in the high‐risk groups (Figure 3D–F). ROC assessments at 1, 2 and 3 years across the validation cohorts further supported the model's favourable prognostic performance (Figure 3G–I). These results indicate that CTLsTKPI offers accurate and stable survival prediction in GBM, supporting clinical risk stratification and informing individualised therapeutic planning.

**FIGURE 3:**
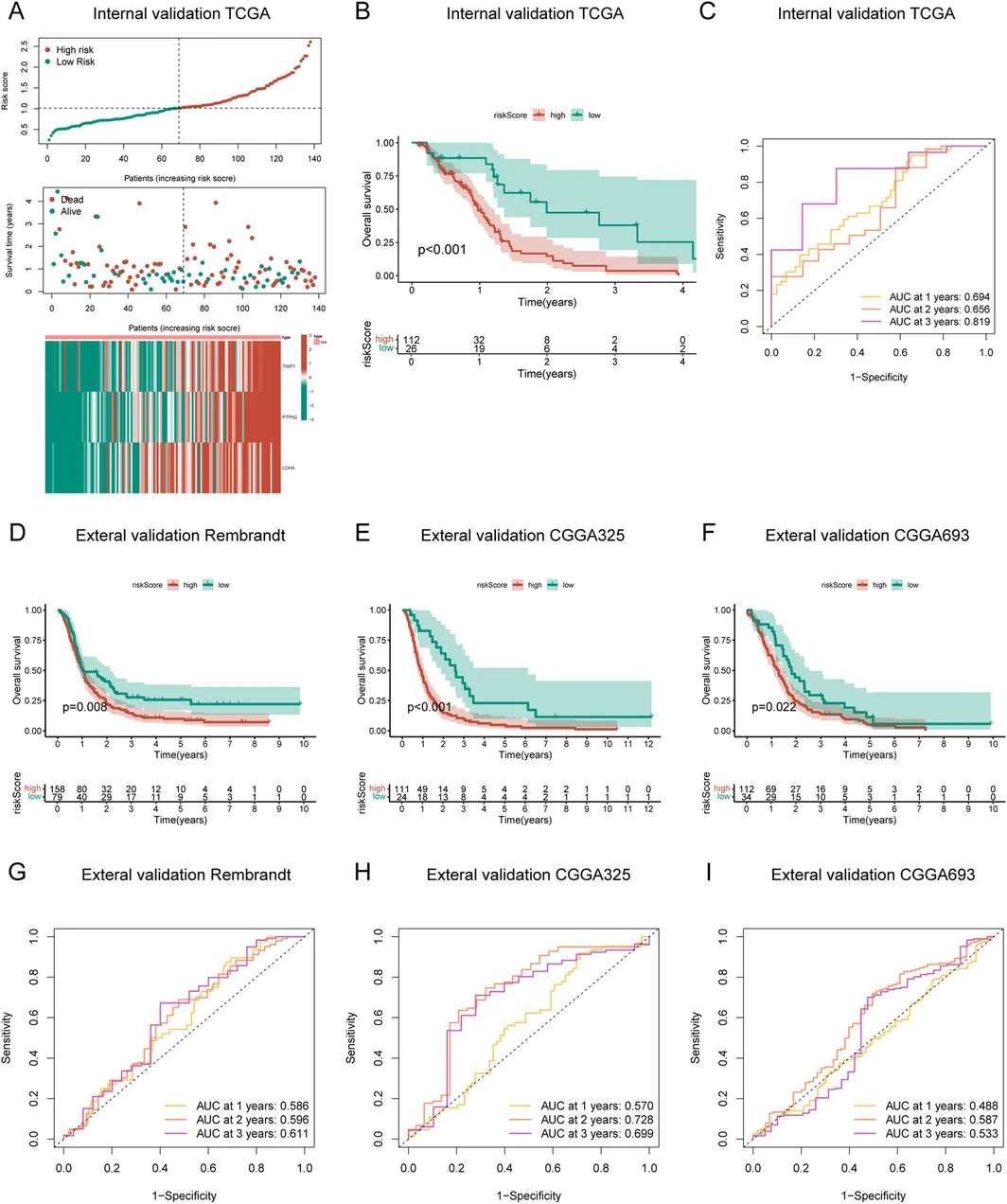
Training and validation of the CTLsTKPI prognostic model. Risk score distribution, survival status and expression of the 3 model genes for GBM patients in the low‐risk and high‐risk groups. (B) Kaplan–Meier analyses in the TCGA cohort stratified by the CTLsTKPI, showing shorter OS in the high‐risk group. (C) ROC curves for 1‐, 2‐ and 3‐year OS in TCGA (AUC = 0.694, 0.656 and 0.819). (D–F) External validation: KM survival analyses in REMBRANDT, CGGA325 and CGGA693 using the same CTLsTKPI formula and cohort‐specific optimal cutpoints, confirming poorer OS in high‐risk patients. (G–I) ROC curves at 1, 2 and 3 years in the REMBRANDT, CGGA325 and CGGA693 cohorts, respectively.

### 3.4 Establish and Assess the Nomogram Model Forecasting Survival

To evaluate the prognostic value of CTLsTKPI, we constructed a multivariate ROC curve based on the TCGA cohort. The results demonstrated that the nomogram model achieved an AUC of 0.78, markedly outperforming other individual predictive factors, including TNIP1 (0.59), PTPN2 (0.60), LDHA (0.66), age (0.66) and sex (0.45) (Figure 4A). Calibration curve analysis confirmed the high predictive accuracy of the model overall survival at 1, 1.5 and 2 years, further supporting its clinical applicability in prognostic assessment (Figure 4B). Finally, we integrated CTLsTKPI with clinicopathological variables using multivariable Cox and stepwise selection to construct the nomogram, which incorporates age, sex, signature gene expression and the CTLsTKPI risk score (Figure 4C).

**FIGURE 4:**
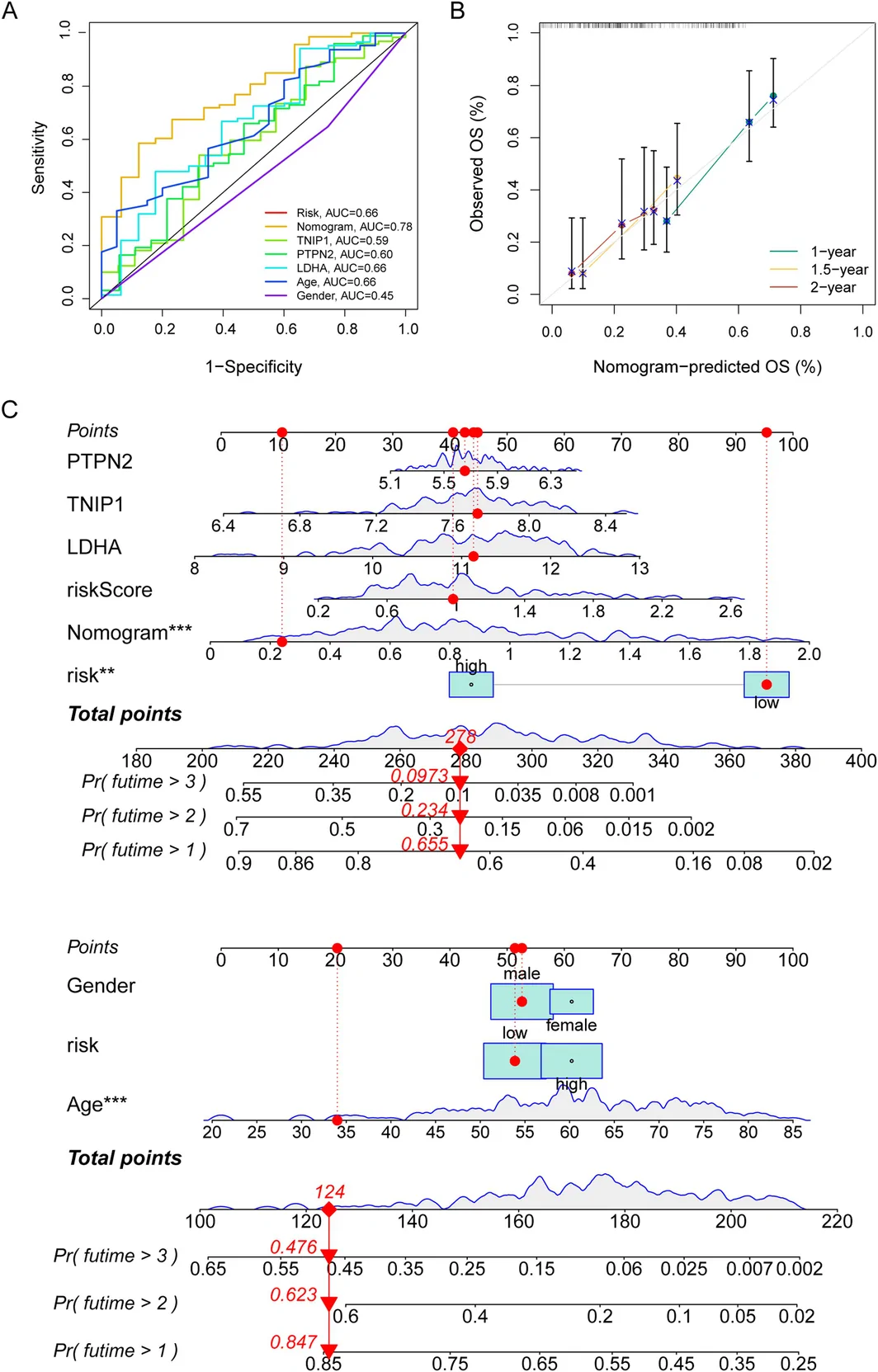
Establishment and assessment of a CTLsTKPI‐based survival prediction nomogram. (A) Multivariate ROC analysis in the TCGA cohort showing superior discrimination of the CTLsTKPI‐based nomogram. (B) Calibration curves demonstrating close agreement between predicted and observed 1‐, 1.5‐ and 2‐year OS. (C) A nomogram determining the prognostic of patients with GBM.*p < 0.05, **p < 0.01, ***p < 0.001, ****p < 0.0001.

In summary, our analyses support CTLsTKPI as a robust and independent prognostic marker in GBM. A multivariable nomogram that integrates CTLsTKPI with key clinicopathologic factors enables individualised survival estimation and may inform evidence‐based clinical decision‐making.

### 3.5 Association of CTLsTKPI With Immune Checkpoints and Tumour Mutation Burden

Using the CTLsTK‐related signature, we derived a risk score that partitioned patients into high‐risk and low‐risk groups and was significantly linked to immune checkpoint activity and tumour mutation burden (TMB). Gene–gene correlation chord analysis indicated that LDHA expression was strongly positively correlated with the glycolytic enzyme PGK1, but inversely correlated with PPM1K, a regulator of branched‐chain amino acid metabolism, consistent with metabolic reprogramming in high‐risk tumours (Figure 5A). Additionally, comparison of immune function and pathway module scores between risk strata showed higher activity in APC_co_inhibition, CCR, checkpoint and T_cell_co_inhibition modules in the high‐risk group (Figure 5B). Concordantly, immune cell–score profiling revealed enrichment of immunosuppressive populations—macrophages and Tregs—in high‐risk tumours (Figure 5C). Moreover, LDHA expression correlated positively with multiple immune checkpoints, including CD274, CTLA4, PDCD1, LAG3 and HAVCR2 (Figure 5D).

**FIGURE 5:**
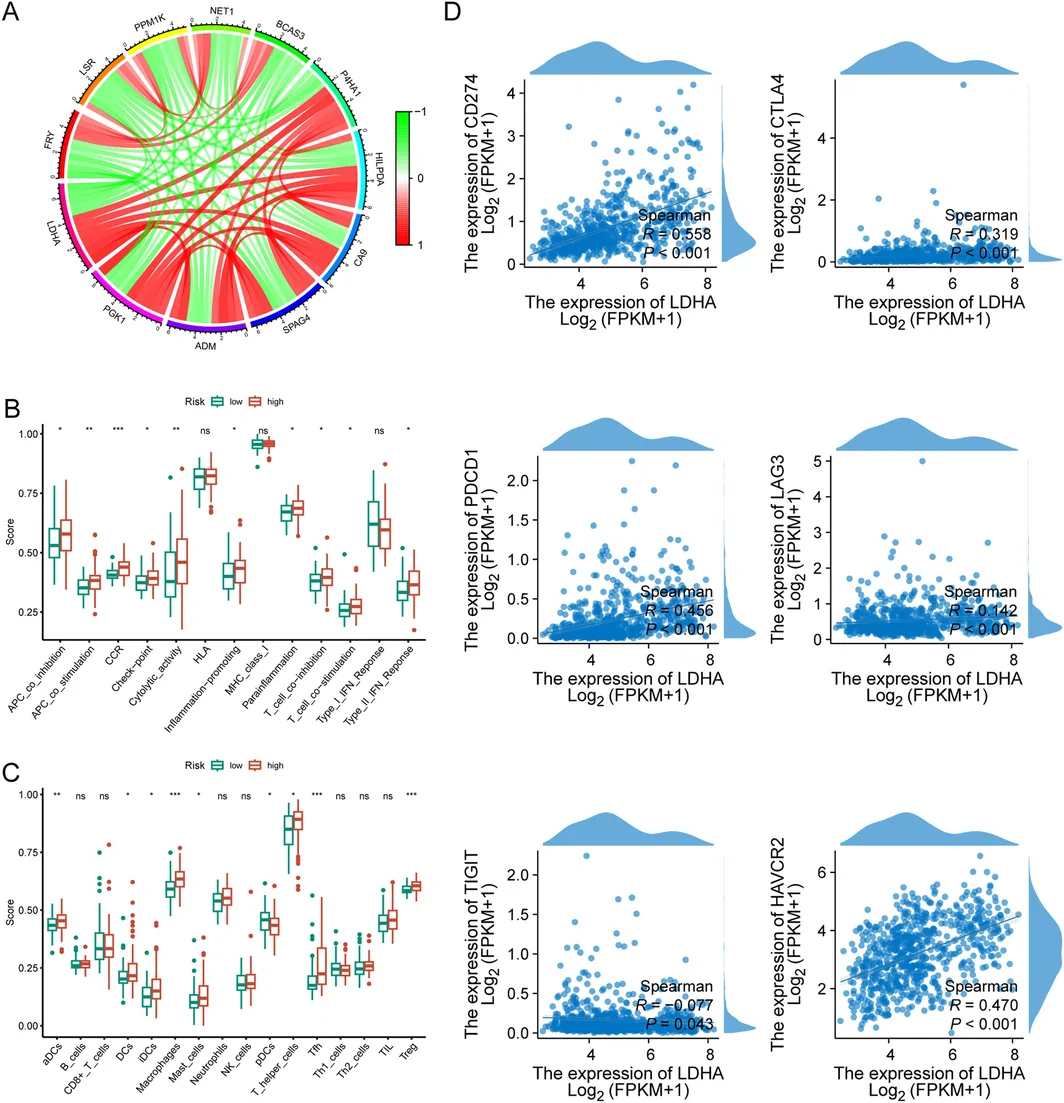
Association of CTLsTKPI with immune checkpoints and tumour mutation burden. (A) Chord plot depicting the LDHA‐associated gene network. (B) Immune function and pathway module scores across risk strata. (C) Immune cell score profiling showing enrichment of macrophages and Tregs in high‐risk tumours. (D) Correlations between LDHA and immune checkpoints (CD274, CTLA4, PDCD1, LAG3, HAVCR2). *p < 0.05, **p < 0.01, ***p < 0.001, ****p < 0.0001.

To further delineate links between the risk score and tumour stemness/TMB, we performed analyses in the TCGA cohort. The risk score showed no meaningful association with DNAss but correlated inversely with RNAss (r = −0.38, *p* = 0.0096), indicating that higher risk does not coincide with greater RNA‐based stemness (Figure S2A,B). Mutation landscapes differed modestly by risk stratum: in the low‐risk group, PTEN, TP53, EGFR and TTN harboured alterations in 31%, 34%, 29% and 31% of cases, respectively, whereas the corresponding frequencies in the high‐risk group were 35%, 35%, 23% and 27% (Figure S2C,D). Survival analyses by TMB demonstrated a benefit for patients with higher mutational load relative to those with lower TMB (Figure S2E). When combining TMB with the risk score, the high‐TMB/low‐risk subgroup exhibited the most favourable outcomes, whereas the low‐TMB/high‐risk subgroup fared worst (Figure S2F).

Taken together, CTLsTKPI appears largely independent of stemness measures yet aligns closely with mutational profiles and TMB, and is associated with an immune‐suppressive checkpoint milieu in GBM. Integrating TMB status with the CTLsTKPI further refines prognostic stratification, underscoring the signature's relevance to GBM immunobiology.

### 3.6 LDHA Expression and Its Association With Immunosuppressive Microenvironment Across Malignancies

To elucidate the role of LDHA in the immune TME of GBM, we examined the association between LDHA expression and immune cell infiltration in TCGA datasets. Both GBM and lower‐grade glioma exhibited a significant inverse association between LDHA expression and NK/CD8^+^ T cells infiltration. (Figure 6A,B). Deconvolution‐based profiling further indicated that tumours with high LDHA were enriched for resting NK cells, accompanied by depletion of activated NK cells, CD8^+^ T cells and M1‐polarised macrophages, suggesting its critical role in shaping an immunosuppressive TME (Figure S3A). Stromal and immune scoring analyses showed that LDHA expression was positively associated with StromalScore, ImmuneScore and ESTIMATEScore, suggesting enrichment of stromal and immune components in tumours with higher LDHA levels (Figure 6C). ROC curve analysis showed that LDHA possessed prognostic predictive capability in GBM, with AUC values of 0.658, 0.606 and 0.702 at 1, 2 and 3 years respectively (Figure 6D). A pan‐cancer survey revealed elevated LDHA expression across multiple malignancies—GBM, COAD, ESCA, HNSC and LUAD—relative to matched normal tissues (Figure 6E). KEGG pathway enrichment of LDHA‐related genes emphasised immune signalling, with prominent involvement of the JAK–STAT and Toll‐like receptor pathways (Figure 6F).

**FIGURE 6:**
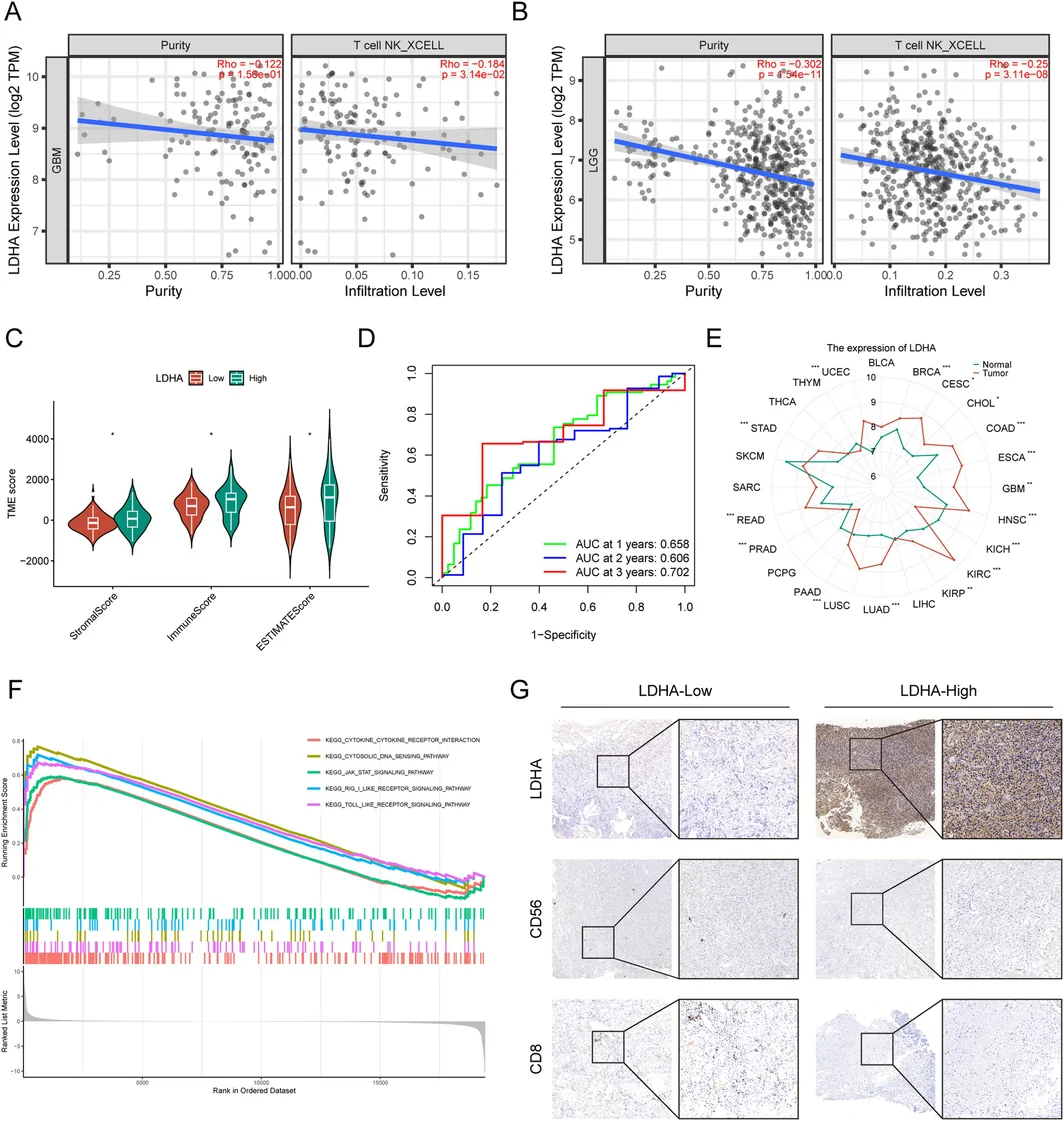
LDHA expression and an immunosuppressive microenvironment across malignancies. (A, B) In TCGA GBM (A) and LGG (B), LDHA expression inversely correlates with T cell NK infiltration. (C) Positive associations between LDHA expression and StromalScore, ImmuneScore and ESTIMATEScore. (D) ROC curves showing prognostic utility of LDHA in GBM. (E) Pan‐cancer analysis indicating elevated LDHA expression in GBM, COAD, ESCA, HNSC and LUAD versus matched normal tissues. (F) KEGG enrichment of LDHA‐related genes, highlighting immune signalling, notably JAK–STAT and Toll‐like receptor pathways. (G) Representative IHC staining of human GBM specimens for LDHA, CD56 and CD8, 100 μm (overview), 25 μm (boxed area).*p < 0.05, **p < 0.01, ***p < 0.001, ****p < 0.0001.

Immunohistochemistry confirmed that low LDHA expression in GBM was associated with increased NK/CD8^+^ T cells infiltration, while high LDHA expression corresponded to their marked reduction (Figure 6G). A similar pattern was observed in colorectal adenocarcinoma (COAD), where adjacent normal tissues exhibited low LDHA expression with abundant NK and CD8^+^ T cells infiltration. Tumours with low LDHA expression showed diminished infiltration, whereas high LDHA tumours were nearly devoid of NK and CD8^+^ T cells (Figure S3B). At a mechanistic level, LDHA expression tracked positively with gene sets involved in glycolysis (Figure S4A), immune regulation (Figure S4B) and cell motility/invasion (Figure S4C). Collectively, these findings indicate that LDHA is highly expressed in GBM and multiple other malignancies, and its upregulation is closely associated with enrichment of immunosuppressive cell populations and depletion of effector immune cells. LDHA may contribute to the establishment of an immunosuppressive TME through glycolytic metabolic reprogramming and immune signalling regulation.

### 3.7 LDHA Promotes Proliferation, Migration and Invasion in GBM Cells

To explore the functional significance of LDHA in GBM, we first compared its protein abundance in tumour tissues and matched peritumoral samples from five patients. LDHA levels were substantially higher in tumour specimens than in adjacent noncancerous tissues (Figure S5A). In addition, A172 and U251 GBM cell lines were selected for further in vitro studies. Stable LDHA‐overexpressing cells were generated via lentiviral transduction, whereas two LDHA‐specific shRNAs were employed to achieve persistent LDHA silencing. RT‐qPCR and Western blot analyses confirmed significant alterations in LDHA expression at both mRNA and protein levels compared with control cells (Figure 7A; Figure S5B,C).

**FIGURE 7:**
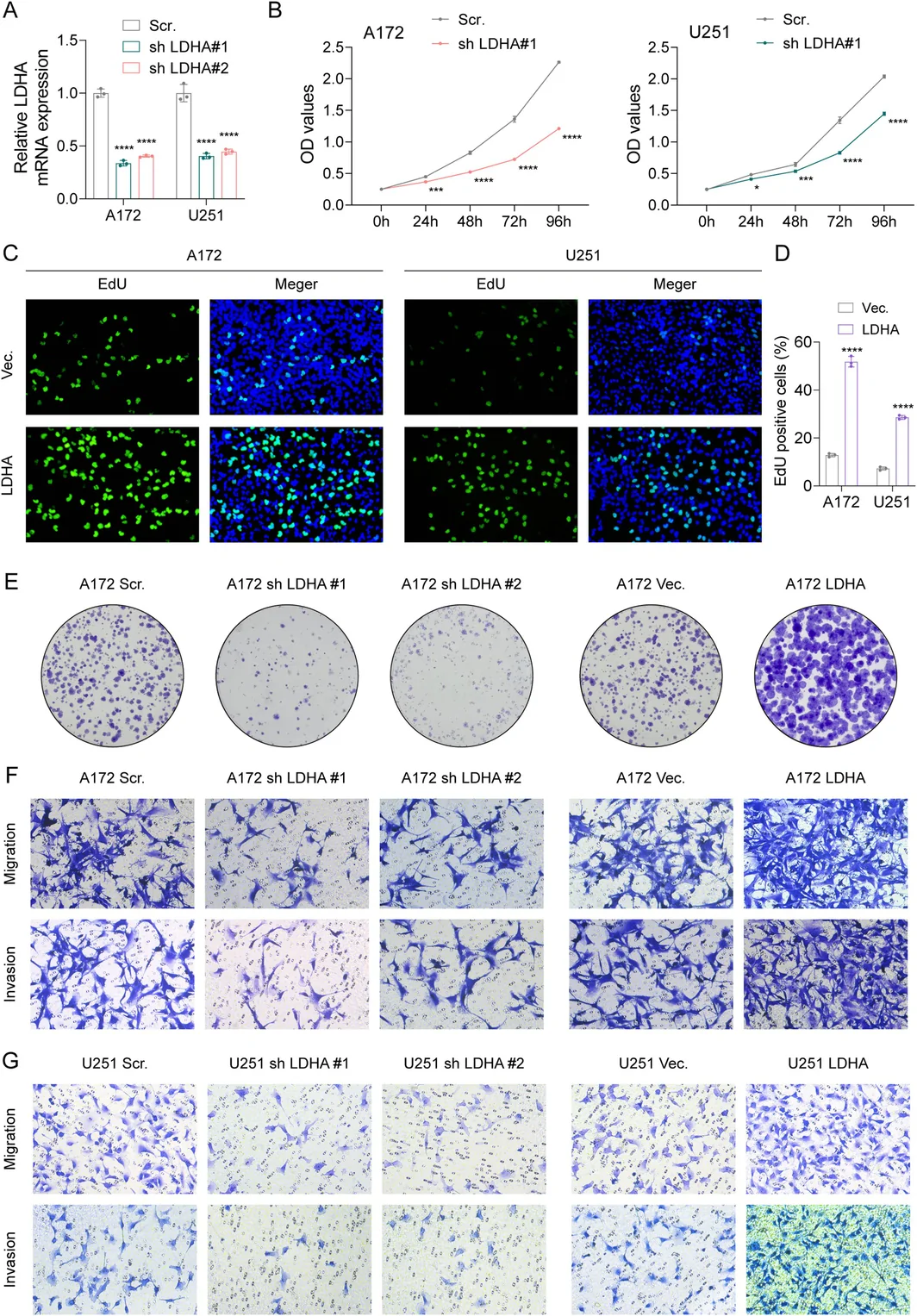
LDHA promotes proliferation, migration and invasion in GBM cells. (A) RT‐qPCR validation of LDHA shRNA‐mediated knockdown in A172 and U251 cells. (B) The growth curves of transfected A172 and U251 cells were determined by CCK8 assays. (C, D) The proliferation of transfected A172 and U251 cells was detected by EdU staining assays. (E) The colony formation assays were performed in transfected A172 and U251 cells. (F, G) The transwell assays showed the migration and invasion abilities of transfected A172 and U251 cells. Data are mean ± SD from *n* = 3 independent experiments; two‐sided Student's *t*‐test.*p < 0.05, **p < 0.01, ***p < 0.001, ****p < 0.0001.

Functional assays demonstrated that LDHA knockdown significantly inhibited the proliferation of A172 and U251 cells, as measured by CCK‐8 assays (Figure 7B). Consistently, EdU incorporation assays revealed a marked reduction in EdU‐positive cells in LDHA‐silenced lines, whereas LDHA overexpression significantly increased the proportion of EdU‐positive cells (Figure 7C,D; Figure S5D–F). Furthermore, our results demonstrate that treatment with the LDHA inhibitor GNE‐140 significantly inhibited the proliferative capacity of GBM cells, further confirming the role of LDHA in promoting tumour growth (Figure S6A,B).

Colony formation assays further confirmed that LDHA overexpression enhanced, while its knockdown suppressed, the clonogenic potential of GBM cells (Figure 7E; Figure S5G). Moreover, Transwell assays demonstrated that LDHA overexpression promoted, while its depletion impaired, the migratory and invasive capacities of GBM cells (Figure 7F,G; Figure S5H,I). As a whole, these findings demonstrate that LDHA exerts oncogenic effects in GBM by promoting cell proliferation, migration and invasion, suggesting a pivotal role in tumour progression. The data in Figure S6C–E, showing the effects of GNE‐140 on cell colony formation, migration and invasion, further confirm the oncogenic role of LDHA in GBM. Additionally, the in vivo data support these findings, showing that LDHA knockdown in the intracranial GL261 glioma model significantly slowed tumour progression and prolonged survival (Figure S7A–C).

### 3.8 LDHA‐Mediated Lactate Accumulation Suppresses CAR‐NK Cell Cytotoxicity and Enhances GBM Immune Evasion

The above results indicated a negative correlation between LDHA expression and cytotoxic lymphocyte infiltration. To further explore whether LDHA contributes to the formation of an immunosuppressive TME in GBM through lactate accumulation, we evaluated the cytotoxic activity of CAR‐NK cells against U251 and A172 cells by flow cytometry. Tumour cells were co‐cultured with CAR‐NK cells at effector‐to‐target (E:T) ratios of 1:3, 1:1 and 3:1, and apoptosis was assessed using PI and Annexin‐V staining. As expected, increasing CAR‐NK cell ratios significantly enhanced tumour cell apoptosis, whereas supplementation with exogenous lactate (10 mM) markedly suppressed CAR‐NK‐mediated cytotoxicity (Figure 8A). Consistently, flow cytometric quantification confirmed that higher E:T ratios promoted cytotoxic efficiency in both U251 and A172 cells, while lactate treatment significantly attenuated this effect (Figure 8B).

**FIGURE 8:**
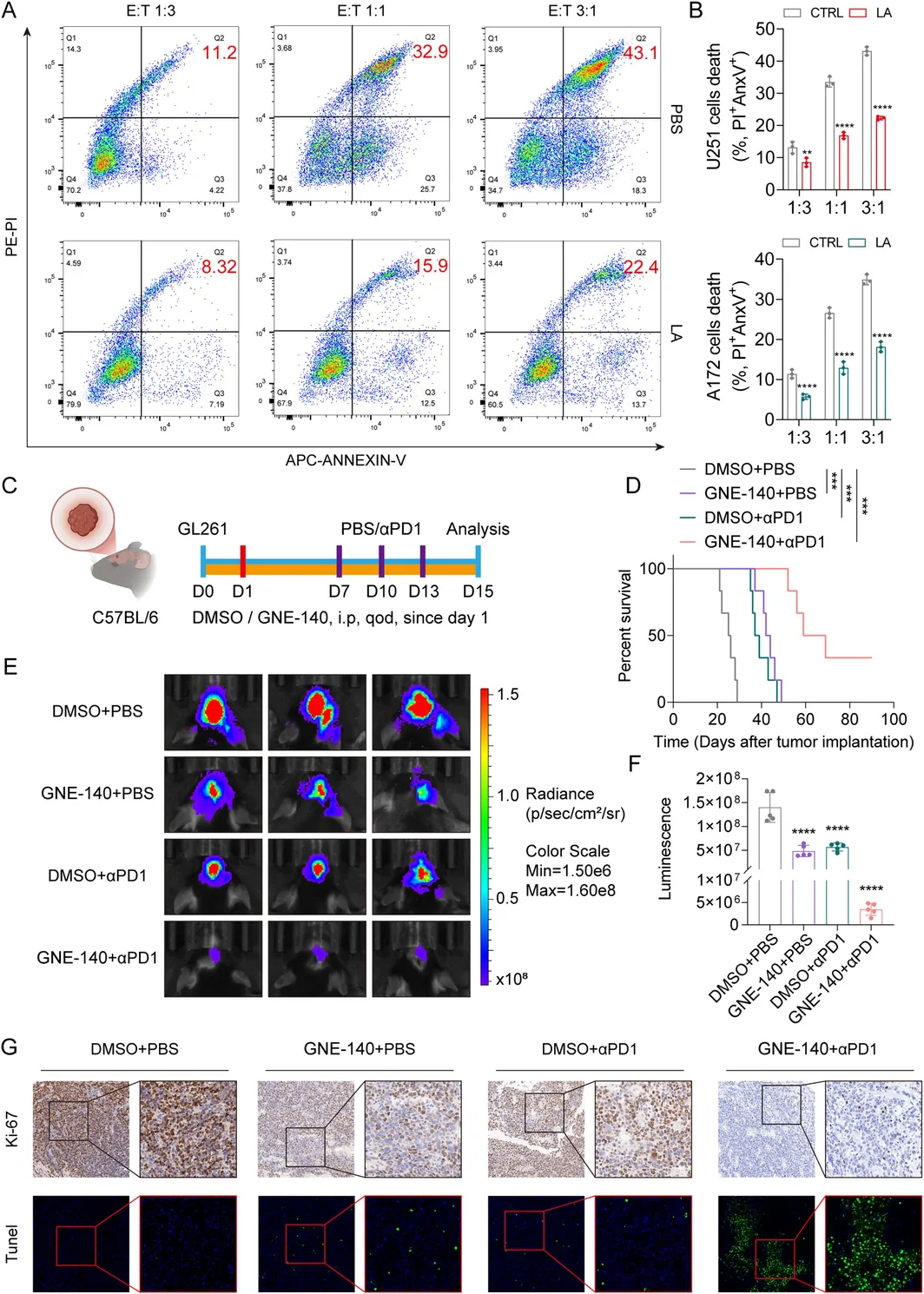
LDHA‐mediated lactate impairs CAR‐NK cytotoxicity and its inhibition enhances immunotherapy in GBM. (A) Flow‐cytometric assessment of apoptosis in U251 and A172 cells co‐cultured with CAR‐NK cells at effector‐to‐target (E:T) ratios of 1:3, 1:1 and 3:1, with or without exogenous lactate (10 mM). (B) Quantification of cytotoxicity across E:T ratios in both cell lines in the presence or absence of lactate. (C) In vivo study schema: GL261 model treated with the LDHA inhibitor GNE‐140 (day 1) and/or anti–PD‐1 (day 7). (D) Kaplan–Meier survival curves for each treatment group. (E‐F) Bioluminescence imaging on day 15 and corresponding quantification of tumour burden. (G) IHC for Ki67 and TUNEL in tumour sections. Data are mean ± SD from *n* = 3 independent experiments; two‐sided Student's *t*‐test. *p < 0.05, **p < 0.01, ***p < 0.001, ****p < 0.0001.

For in vivo validation, we established an orthotopic GL261 GBM model. Mice were administered the LDHA inhibitor GNE‐140 beginning on day 1 after tumour implantation, followed by PD‐1 antibody treatment initiated on day 7. Animals were randomised into four groups: DMSO+PBS, GNE‐140 + PBS, DMSO+αPD‐1 and GNE‐140 + αPD‐1 (Figure 8C). Kaplan–Meier analysis demonstrated that the combination of GNE‐140 and αPD‐1 significantly extended survival compared with the other groups (Figure 8D). Bioluminescent imaging (BLI) further showed a marked reduction in tumour volume by day 15 in the combination therapy cohort, with statistically significant differences relative to controls (Figure 8E,F). Consistently, IHC staining for Ki67 and TUNEL confirmed that combined treatment effectively suppressed tumour cell proliferation while enhancing apoptosis within tumour tissues (Figure 8G). All in all, these findings indicate that LDHA drives the formation of an immunosuppressive TME in GBM through lactate accumulation. Pharmacological inhibition of LDHA augments the therapeutic efficacy of immunotherapy and markedly prolongs survival, providing a strong mechanistic basis for targeting LDHA as a strategy to improve GBM immunotherapy.

## 4 Discussion

Building on CTLsTK as an immune reference, we systematically profiled DEGs associated with NK/CD8^+^ T cells infiltration in GBM and derived a three‐gene signature (LDHA, TNIP1, PTPN2) with a composite risk score (CTLsTKPI). This score consistently stratified patients into high‐ and low‐risk groups across independent cohorts and, when integrated with clinicopathologic variables in a nomogram, further improved individualised survival prediction. CTLsTKPI strata also aligned with immune‐checkpoint activity and TMB, providing a biologically grounded framework for selecting patients more likely to benefit from immunotherapy.

Consistent with these observations, high‐risk tumours exhibit heightened checkpoint signalling and enrichment of immunosuppressive populations (macrophages and Tregs), with concomitant depletion of activated NK and CD8^+^ T cells, which helps explain resistance to immunotherapy in GBM [33]. Although CTLsTKPI did not correlate with DNA‐based stemness (DNAss), it associated negatively with RNA‐based stemness, suggesting that transcriptional reprogramming rather than fixed genomic changes underlie its prognostic effect. Combining CTLsTKPI with TMB improved risk discrimination, indicating value in integrating transcriptional and mutational axes [34]. In parallel, extensive evidence links aberrant glycolysis to stemness, invasiveness and poor outcomes across cancers [35, 36]. Within this pathway, LDHA (the pyruvate‐to‐lactate catalyst) has emerged as a central target; its overexpression associates with poor survival and resistance to chemo‐ and radiotherapy [37, 38, 39].

Within our model, LDHA was the dominant contributor to adverse survival and inversely associated with cytotoxic lymphocyte infiltration [40]. Mechanistically, elevated glycolysis drives LDHA‐mediated lactate production under HIF‐1 regulation, regenerating NAD^+^ and promoting lactate export via monocarboxylate transporters, which acidifies the TME and impairs effector lymphocyte function [41, 42, 43, 44]. Correlative analyses showed LDHA positively tracks with PGK1 and negatively with the branched‐chain amino‐acid regulator PPM1K, consistent with reinforced glycolysis and metabolic reprogramming. LDHA expression also correlated with higher Stromal/Immune/ESTIMATE scores and was broadly upregulated pan‐cancer; enrichment analyses implicated JAK–STAT and Toll‐like receptor signalling, pointing to a glycolysis‐lactate axis that fosters immune exclusion [45]. Functional assays were concordant: LDHA overexpression enhanced GBM cell proliferation, clonogenicity and invasion, whereas LDHA silencing produced the opposite effects. As rapidly dividing cancer cells increase glycolytic flux, excess lactate acidifies the extracellular milieu, promoting invasion, angiogenesis, metastasis and immune suppression [46, 47, 48]. Collectively, LDHA operates not merely as a metabolic enzyme but as a central node linking metabolic reprogramming to immune evasion in GBM [49, 50].

Consistent with this model, LDHA‐driven lactate accumulation also blunts CAR‐NK cytotoxicity, reinforcing immune suppression. In vitro, raising the effector‐to‐target ratio increased tumour apoptosis, yet exogenous lactate markedly reduced CAR‐NK killing. In vivo, pharmacologic LDHA inhibition (e.g., GNE‐140), particularly with PD‐1 blockade, curtailed tumour growth and prolonged survival in an orthotopic GBM model; immunohistochemistry under combination therapy showed reduced proliferation and increased apoptosis [51]. These findings indicate that targeting LDHA can sensitise GBM to immunotherapy and partially overcome intrinsic resistance to checkpoint inhibition. Similarly, loss of LDHA function in melanoma increased NK and CD8^+^ T cells infiltration with higher IFN‐γ, supporting immune‐microenvironment reprogramming and improved checkpoint efficacy [52, 53]. At the effector level, lactate suppresses IFN‐γ, granzyme B and perforin and impedes NK and CD8^+^ T cells proliferation, collectively weakening antitumour responses [20, 54].

From a translational medicine perspective, CTLsTKPI shows promise as an independent prognostic biomarker and as a practical tool to enrich candidates for cellular immunotherapy and immune‐checkpoint inhibitors. Nevertheless, several limitations merit consideration. Our conclusions are grounded in transcriptomic inference with supporting in vitro and in vivo data; bulk deconvolution requires cross‐validation using single‐cell and spatial transcriptomics plus IHC. In addition, preclinical models fail to fully recapitulate human GBM, particularly the blood–brain barrier, the depth of immunosuppression and intratumoral heterogeneity; therefore, prospective clinical studies are required. Moreover, LDHA inhibitors require optimisation for brain penetration, target selectivity and therapeutic window before clinical deployment.

In conclusion, our data position LDHA as both a prognostic biomarker and a therapeutic target that drives metabolic reprogramming and sculpts an immunosuppressive TME to promote tumour progression and immune escape [55]. Targeting LDHA represents a rational strategy to enhance immunotherapy in GBM. Prospective clinical trials are warranted to validate these findings and to establish the safety, pharmacodynamics and clinical benefit of incorporating LDHA inhibitors into combination regimens.

## 5 Conclusions

Using CTLsTK as an immune anchor, we derived a three‐gene signature (LDHA, TNIP1, PTPN2) and risk score (CTLsTKPI) that consistently stratifies GBM and improves nomogram‐based survival prediction. CTLsTKPI mirrors the immune milieu and complements TMB for immunotherapy selection. LDHA emerged as the dominant adverse node: heightened glycolytic flux and lactate export drove immune exclusion, while pharmacologic LDHA blockade restored CAR‐NK activity, enhanced PD‐1 efficacy and curtailed tumour growth in vivo. Thus, LDHA is both a biomarker and a target. Single‐cell/spatial validation and brain‐penetrant LDHA inhibitors should precede biomarker‐guided trials pairing LDHA blockade with checkpoint or cellular therapies.

## Author Contributions

Shaojie Yu and Xudong Li designed and wrote the manuscript. Shaojie Yu, Xudong Li and Ji Wu helped perform the in vitro and in vivo experiments. Minjie Wang, Zihan Gong and Baowen Zhang performed IHC staining of patient tumour samples. Shaojie Yu, Xudong Li, and Ji Wu conducted data analysis. Haojun Shi, Xuan Wang, and Wei Xiang directed the research.

## Funding

This work was supported by the Open Foundation of Hubei Key Laboratory of Biological Targeted Therapy (202414) and National Outstanding Youth Science Fund Project of the National Natural Science Foundation of China, 82203140.

## Conflicts of Interest

The authors declare no conflicts of interest.

## Supporting information


**Figure S1:** Risk stratification across external cohorts.
**Figure S2:** Stemness, mutation landscape and TMB stratification by CTLsTKPI.
(A, B) Scatterplots of CTLsTKPI versus stemness indices (DNAss and RNAss). (C, D) Oncoplots of somatic mutation profiles in low‐risk and high‐risk groups, with selected genes (PTEN, TP53, EGFR, TTN) and their mutation frequencies. (E) Kaplan–Meier survival curves stratified by tumour mutation burden (high vs. low TMB). (F) Kaplan–Meier survival curves stratified jointly by TMB (high vs. low) and CTLsTKPI risk (high vs. low).
**Figure S3:** Deconvolution‐based immune infiltration analysis and COAD IHC.
(A) Deconvolution profiles of immune cell subsets in LDHA‐high and LDHA‐low tumours, displaying resting NK cells, activated NK cells, CD8^+^ T cells and M1 macrophages. (B) Representative IHC staining of human COAD specimens and adjacent normal tissue for LDHA, CD56 and CD8, 100 μm (overview), 25 μm (boxed area).
**Figure S4:** Pathway and gene‐set associations of LDHA.
(A‐C) Gene‐set enrichment analyses showing enrichment of glycolysis (A), immune‐regulatory programmes (B) and cell motility/invasion pathways (C).
**Figure S6:** Validation of GNE‐140 inhibitor effects on proliferation, migration and invasion in GBM cells.
**Figure S7:** Effect of LDHA knockdown on tumour progression and survival in an intracranial glioma model.

**Figure S5:** Validation of LDHA modulation and extended functional assays.
(A) LDHA protein levels in GBM tumours and adjacent normal tissues (*n* = 5 pairs). (B, C) Western blots of LDHA in A172 and U251 cells with shRNA‐mediated knockdown or overexpression. (D, F) EdU staining in transfected A172 and U251 cells. (G) Colony formation assay in transfected U251 cells. (H, I) Quantification of Transwell assays for transfected A172 (H) and U251 (I) cells. Data are mean ± SD from *n* = 3 independent experiments; two‐sided Student’s *t*‐test.

**Table S1:** Legend. Cytotoxic lymphocyte–mediated tumour‐killing (CTLsTK)‐related genes identified by immune infiltration correlation analysis. Table S1 Cytotoxic lymphocyte–mediated tumour‐killing (CTLsTK)‐related genes identified by immune infiltration correlation analysis.

## Data Availability

The data that support the findings of this study are available from the corresponding author upon reasonable request.
