# Supplementary material for: Integrative Analysis of Cytotoxic Lymphocytes‐Mediated Tumour Killing Related Genes Reveals LDHA as a Novel Glioblastoma Therapeutic Target
Source: J Cell Mol Med. 2026 Feb 13;30(4):e71054. doi: 10.1111/jcmm.71054 (PMC13749616; doi:10.1111/jcmm.71054)

Supplementary Fig. 5

LDHA

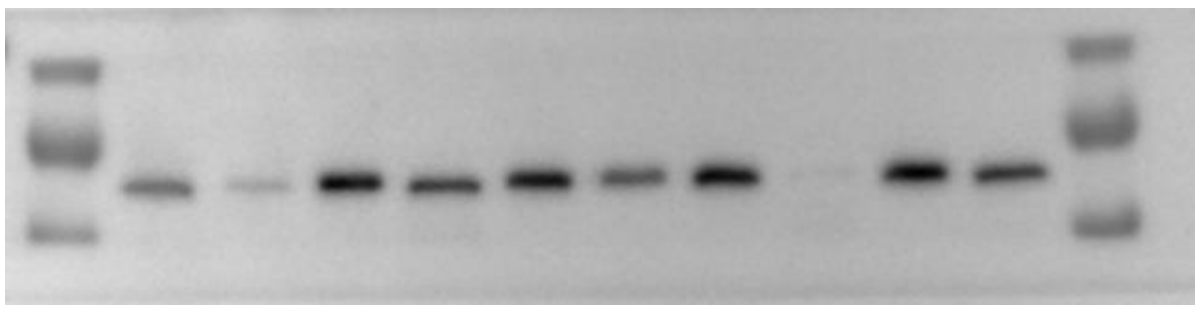

LDHA

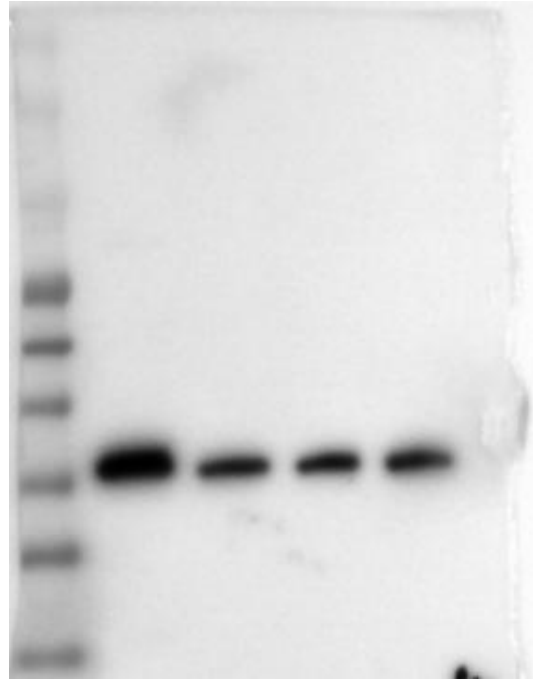

GAPDH

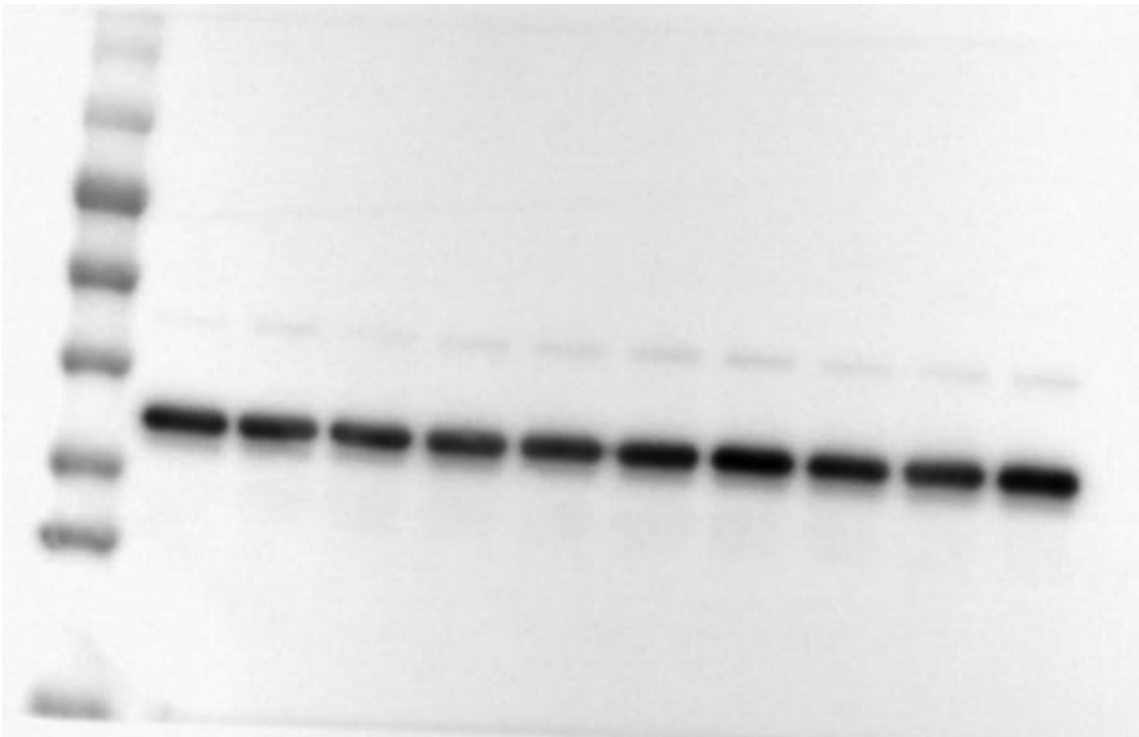

GAPDH

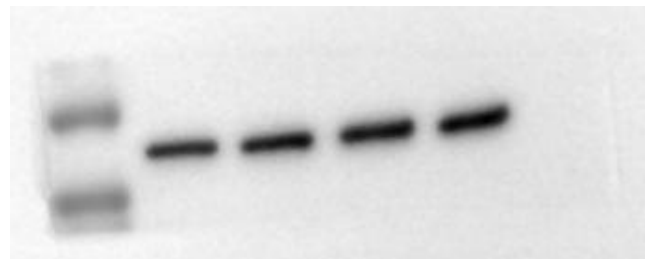

LDHA

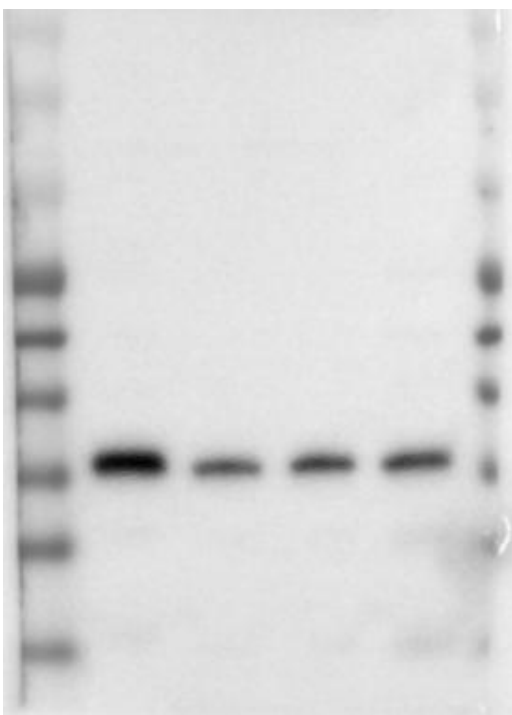

LDHA

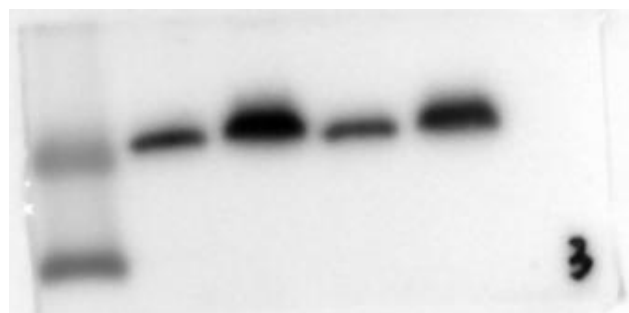

GAPDH

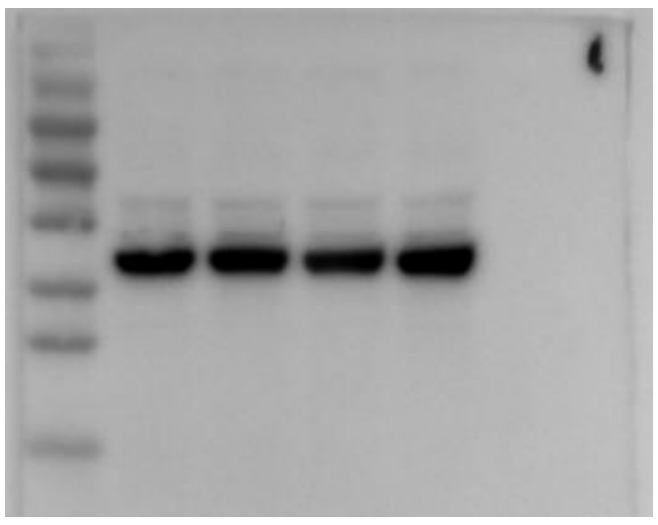

GAPDH

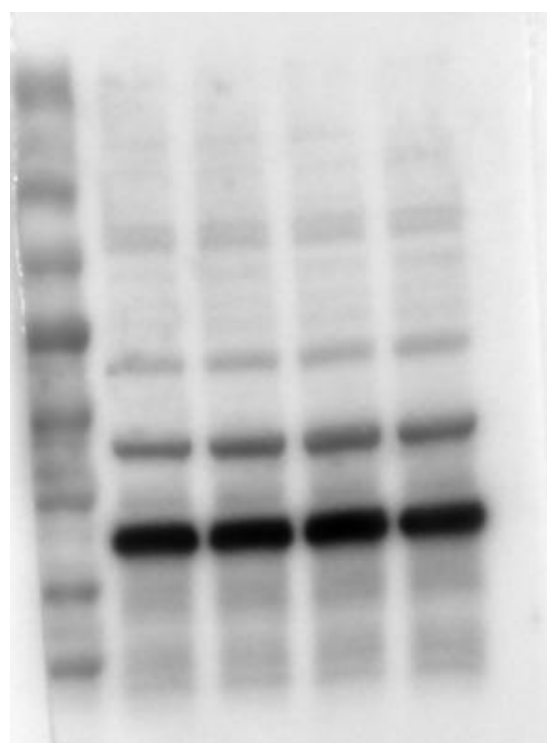

Supplement: Supplementary file 1 [file JCMM-30-e71054-s001.pdf]
