# Supplementary material for: Integrative Analysis of Cytotoxic Lymphocytes‐Mediated Tumour Killing Related Genes Reveals LDHA as a Novel Glioblastoma Therapeutic Target
Source: J Cell Mol Med. 2026 Feb 13;30(4):e71054. doi: 10.1111/jcmm.71054 (PMC13749616; doi:10.1111/jcmm.71054)
Supplement: Supplementary file 2 [file JCMM-30-e71054-s003.docx]

**Supplementary Fig. 1 Risk stratification across external cohorts**

(A-C) CTLsTKPI-based risk score distribution, survival status, and three-gene CTLsTK signature expression (LDHA, TNIP1, PTPN2) in REMBRANDT (A), CGGA325 (B), and CGGA693 (C), respectively.

**Supplementary Fig. 2 Stemness, mutation landscape, and TMB stratification by CTLsTKPI.**
(A-B) Scatterplots of CTLsTKPI versus stemness indices (DNAss and RNAss). (C-D) Oncoplots of somatic mutation profiles in low-risk and high-risk groups, with selected genes (PTEN, TP53, EGFR, TTN) and their mutation frequencies. (E) Kaplan-Meier survival curves stratified by tumor mutation burden (high vs. low TMB). (F) Kaplan-Meier survival curves stratified jointly by TMB (high vs. low) and CTLsTKPI risk (high vs. low).

**Supplementary Fig. 3 Deconvolution-based immune infiltration analysis and COAD IHC.**
(A) Deconvolution profiles of immune cell subsets in LDHA-high and LDHA-low tumors, displaying resting NK cells, activated NK cells, CD8⁺ T cells, and M1 macrophages. (B) Representative IHC staining of human COAD specimens and adjacent normal tissue for LDHA, CD56 and CD8, 100μm (overview), 25μm (boxed area).

**Supplementary Fig. 4 Pathway and gene-set associations of LDHA.**
(A-C) Gene-set enrichment analyses showing enrichment of glycolysis (A), immune-regulatory programs (B), and cell motility/invasion pathways (C).

**Supplementary Fig. 5 Validation of LDHA modulation and extended functional assays.**
(A) LDHA protein levels in GBM tumors and adjacent normal tissues (n = 5 pairs). (B-C) Western blots of LDHA in A172 and U251 cells with shRNA-mediated knockdown or overexpression. (D-F) EdU staining in transfected A172 and U251 cells. (G) Colony formation assay in transfected U251 cells. (H–I) Quantification of Transwell assays for transfected A172 (H) and U251 (I) cells. Data are mean ± SD from n = 3 independent experiments; two-sided Student’s t-test.

**Supplementary Fig. 6. Validation of GNE-140 inhibitor effects on proliferation, migration, and invasion in GBM cells.**

(A) EdU staining in A172 and U251 cells treated with DMSO or GNE-140 inhibitor (300μM). (B) Quantification of EdU staining in treated A172 and U251 cells. (C) Colony formation assays in A172 and U251 cells with DMSO or GNE-140 inhibitor. (D) Transwell assays showing migration and invasion in treated A172 and U251 cells. (E) Quantification of migration and invasion in A172 and U251 cells. Data are mean ± SD from n = 3 independent experiments; two-sided Student’s t-test.

**Supplementary Fig. 7. Effect of LDHA knockdown on tumor progression and survival in an intracranial glioma model.**

(A) Bioluminescence imaging (BLI) assessment of intracranial tumor growth in Luc-GL261 tumors with LDHA knockdown. (B) Kaplan-Meier survival curves for the LDHA knockdown and control groups. (C) Quantification of fluorescence intensity from BLI imaging. Data are mean ± SD from n = 5 mice per group; two-sided Student’s t-test.
